# Programme evaluation training for health professionals in francophone Africa: process, competence acquisition and use

**DOI:** 10.1186/1478-4491-7-3

**Published:** 2009-01-15

**Authors:** Valéry Ridde, Pierre Fournier, Baya Banza, Caroline Tourigny, Dieudonné Ouédraogo

**Affiliations:** 1 Centre de recherche du Centre Hospitalier de l'Université de Montréal, Montréal, Canada; 2 Department of Social and Preventive Medicine, University of Montreal, Montréal, Canada; 3 Institut Supérieur des Sciences de la Population, Université de Ouagadougou, Ouagadougou, Burkina Faso

## Abstract

**Background:**

While evaluation is, in theory, a component of training programmes in health planning, training needs in this area remain significant. Improving health systems necessarily calls for having more professionals who are skilled in evaluation. Thus, the Université de Ouagadougou (Burkina Faso) and the Université de Montréal (Canada) have partnered to establish, in Burkina Faso, a master's-degree programme in population and health with a course in programme evaluation. This article describes the four-week (150-hour) course taken by two cohorts (2005–2006/2006–2007) of health professionals from 11 francophone African countries. We discuss how the course came to be, its content, its teaching processes and the master's programme results for students.

**Methods:**

The conceptual framework was adapted from Kirkpatrick's (1996) four-level evaluation model: reaction, learning, behaviour, results. Reaction was evaluated based on a standardized questionnaire for all the master's courses and lessons. Learning and behaviour competences were assessed by means of a questionnaire (pretest/post-test, one year after) adapted from the work of Stevahn L, King JA, Ghere G, Minnema J: Establishing Essential Competencies for Program Evaluators. *Am J Eval *2005, 26(1):43–59. Master's programme effects were tested by comparing the difference in mean scores between times (before, after, one year after) using pretest/post-test designs. Paired sample tests were used to compare mean scores.

**Results:**

The teaching is skills-based, interactive and participative. Students of the first cohort gave the evaluation course the highest score (4.4/5) for overall satisfaction among the 16 courses (3.4–4.4) in the master's programme. What they most appreciated was that the forms of evaluation were well adapted to the content and format of the learning activities. By the end of the master's programme, both cohorts of students considered that they had greatly improved their mastery of the 60 competences (p < 0.001). This level was maintained one year after completing the master's degree, except for reflective practice (p < 0.05). Those who had carried out an evaluation in the intervening 12 months reported a negative gap between their declared mastery and their actual application. However, this is only statistically significant for reflective practice (p < 0.05).

**Conclusion:**

This study shows the importance of integrating summative evaluation into the learning process. Skills-based teaching is much appreciated and well-adapted. Creating a master's programme in population and health in Africa and providing training in evaluation to high-level health professionals from many countries augurs well for scaling up the practice of evaluation in African health systems.

## Background

Obtaining international funding in health care is becoming increasingly competitive. For example, to acquire resources needed to fight HIV/AIDS or malaria, African countries must now participate in Global Fund competitions. This situation presents health care managers with two new challenges. First, their requests and action plans increasingly need to be evidence-based. Managers therefore must be able to understand and assess the quality of data and of intervention evaluations. The second challenge is that, when assessing requests, funding agencies look at how well previously-funded programmes met their objectives. These programmes' effectiveness must therefore be demonstrated. Health care managers can no longer be just good planners. They also must be informed evaluators, or have at least the basic knowledge required to interact effectively with the evaluation experts whom they will recruit. Within the current trend of establishing New Public Management in health care in developing countries [1] and the Paris Declaration on Aid Effectiveness [2], programme evaluation will become a major sphere of activity for the coming decade. Yet programme evaluation is rarely addressed in training programmes for health planning [3] and, in Africa, evaluation processes are still too often imposed by external bodies [4].

A series of regional seminars on evaluation planned by the Development Assistance Committee of the Organisation for Economic Co-operation and Development (OECD) was started in 1990 in Côte d'Ivoire [5]. In 1999, the African Evaluation Association (AfrEA) was launched. Despite these efforts, training in programme evaluation remains a relative rarity on the African continent. There are some seminars and workshops, but few training programmes leading to degrees. This is particularly true in francophone Africa [6,7]. The strengthening of evaluation capacity building (ECB) has thus become an urgent matter in Africa. Experts in this field are asking for more empirical case studies to document the range of practices in order to improve their knowledge [8,9], as ECB is "an emergent field of practice" [10]. University training is one useful strategy for ECB [11]. A review of articles published in this field between 1965 and 2003 reveals a lack of literature on practical evaluation training [12]. This article presents the evaluation of a course on programme evaluation, a four-week (150-hour) course attended by health professionals from 11 francophone African countries.

The Université de Ouagadougou (Burkina Faso) and the Université de Montréal (Canada) have partnered to establish, in Burkina Faso, a master's-degree programme in population and health that includes a course in evaluation. This master's programme is part of a larger programme aimed at reinforcing human and institutional capacities in the analysis and evaluation of public policies and programmes. Its goal is to offer a credible alternative to training programmes offered in North America and Europe. The master's-level training programme includes 12 months of course work and a three-month internship (Table 1). The training is organized into modules of several consecutive days, to accommodate the teachers who are brought in from a number of African countries and from Canada.

**Table 1:** Structure of the master's programme in 2005–2006

**Blocks and modules**	**Course titles**	**Number of lessons and %**
BLOCK 1	Fundamentals and issues in population and health	31 (16%)

Module 1.1	Fundamentals	19

Module 1.2	Issues	12

BLOCK 2	Analysis of population and health issues	62 (31%)

Module 2.1	Introduction to empirical research methodology	4

Module 2.2	Quantitative data sources and basic descriptive statistics	8

Module 2.3	Elements of demography and epidemiology	20

Module 2.4	Elements of qualitative analysis	14

Module 2.5	Introduction to multivariate statistical methods	16

BLOCK 3	Analysis of policies and intervention strategies	48 (23%)

Module 3.1	Fundamentals of public policy analysis	16

Module 3.2	Analysis of health systems	13

Module 3.3	Evaluation	19

BLOCK 4	Communication	13 (7%)

Module 4.1	Communication as a tool for influencing individual and collective health and well-being	7

Module 4.2	Advocacy and information management	6

BLOCK 5	Planning and implementation of interventions	47 (23%)

Module 5.1	Principles of planning for interventions	9

Module 5.2	Operational planning of interventions (programmes, projects)	17

Module 5.3	Implementation of interventions: resource management	13

Module 5.4	Monitoring interventions	8

BLOCK 6	Internship	(3 months)

**TOTAL**		**201**

The overall objective of the master's programme is to develop students' knowledge and aptitudes in analysis, formulation and implementation management, as well as in the evaluation of population and health programmes, including a specific course in programme evaluation (Table 1: 3.3). Before presenting the results achieved in the master's programme, we will describe how the course was implemented and its content related to evaluation.

### The programme evaluation course: process and content

The entire content of the master's programme was planned between 2003 and 2004. The organization into modules and the content of each module were decided using a participative process, after an inventory of training programmes in population and health in several francophone African countries [13]. Because the evaluation course integrates all the knowledge and competences acquired in the other courses, it was positioned as the last course taken by students at the end of the 12 months (Table 1). Teaching, when required, would be carried out by African-Canadian pairs, based on the partnership model [14,15]. The course content took into account:

• the competences expected of programme evaluators [16,17];

• the training needs in evaluation in Africa [6,7];

• prior experience of training in evaluation;

• familiarity with training needs of African students.

**Figure 1:**
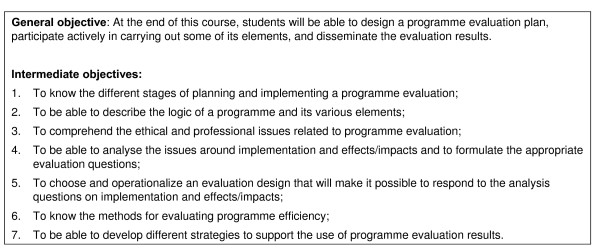
**Course objectives**.

After this process, the teaching objectives (Fig. 1) and course content (Table 2) were finalized.

**Table 2:** Lessons of the programme evaluation course for Cohort 1

Lesson 3.3.1	Introduction and overall process for evaluation
Lesson 3.3.2	Types and approaches to evaluation

Lesson 3.3.3	Intervention logic

Lesson 3.3.4	Standards and practices in evaluation

	*Formative evaluation*

Lesson 3.3.5	Implementation analysis 1 (conceptual bases)

Lesson 3.3.6	Implementation analysis 2 (case study: step 1)

Lesson 3.3.7	Implementation analysis 3 (case study: step 2)

Lesson 3.3.8	Analysis of effects/impact 1 (causal attribution)

Lesson 3.3.9	Evaluation design 1: principles and practices

Lesson 3.3.10	Evaluation design 2 (case study: step 3)

Lesson 3.3.11	Efficiency 1

Lesson 3.3.12	Efficiency 2

Lesson 3.3.13	Utilization of evaluation results

Lesson 3.3.14	Group work and consultation sessions

Lesson 3.3.15	Group work

Lesson 3.3.16	Group work and consultation sessions

Lesson 3.3.17	Group work

	*Summative evaluation*

The aim is to train professionals who will be able to design, support or carry out a programme evaluation. Students are expected to write an evaluation plan. The course involves 19 lectures or sessions (9.5% of total), corresponding to 147 hours of work:

• 52 hours in class in 13 course sessions;

• 52 hours of individual preparatory work;

• 35 hours of group work in preparing evaluations;

• 8 hours of presence for evaluations.

This approach represents a departure from classical teaching methods that generally involve lectures and sometimes directed work. In fact, such methods are rarely effective in training programmes for health personnel in low-income countries [14]. In the case presented here, the entire process is centred on active training in which the student's learning is encouraged, professional experience is validated and course content is more practical than theoretical. Learners actively construct knowledge in collaborative groups [18]. The course uses a myriad of teaching approaches (Table 3) based our own experiences as well as well on the literature [19-22], from which some exercises were adapted (see additional files 1 and 2).

**Table 3:** Examples of pedagogical techniques

**Technique**	**Content**	**Description**
Simulation	Ethical dilemma of negotiation with a client	A client asks an evaluator to change his evaluation design to please a funding agency. Two groups of students receive the same case description; one is in favor of the request, and the other against.

Drawing	Definition of the evaluation	Each student must produce a drawing representing his or her perception of the evaluation (see additional file 1)

	Logic model	Each team of students must prepare a graphic representation of the constituent elements of a programme's logic (see additional file 2)

Case study	Theory of intervention	Each team must determine the theory of the intervention with its various components based upon a real case: the referral system for obstetrical emergencies at Kayes in Mali.

Problem-based learning	Evaluation plan	Each team must draw up an evaluation plan for a health district in Burkina Faso.

Debate	Evaluation profession	A senior public health consultant is invited to discuss with the class the profession in Africa. Students must prepare questions in advance.

Methods for evaluating students provide an opportunity to improve their knowledge and competences in a two-step learning process. First, a formative evaluation (20% of the final course grade) is organized after the first four lessons of the course (Table 2), which constitute a general introduction to programme evaluation. At the end of this first block, groups of four students are given a day-and-a-half to prepare an oral presentation of a draft evaluation plan. Each team receives the plan for a Burkina Faso health district (a real case), selects a specific theme (AIDS, maternal health, etc.), and then develops and presents the draft of its evaluation plan. This presentation allows the teachers to verify the level of understanding of concepts and whether the evaluation plan is on track. Before the presentation, students also have several occasions to receive feedback on their learning.

**Figure 2:**
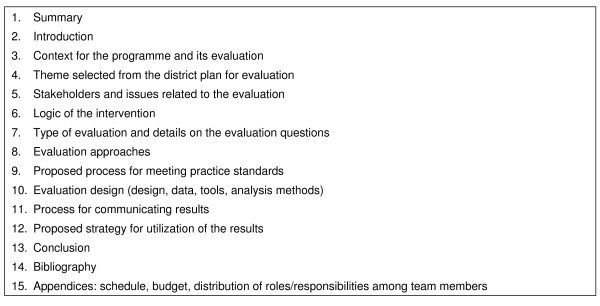
**Contents of an evaluation plan**.

Later, a summative evaluation (80% of the final grade) takes place at the end of the course. Communication skills are also evaluated. Students are expected to write a complete evaluation plan based on the elements presented in Fig. 2.

Knowledge acquired in the course is thus integrated in this final project, which is presented orally. Peers have the opportunity to ask questions and give feedback on their colleagues' work. Students are given four days to carry out this project, during which each group has two one-hour consultation sessions with the teacher.

We present here the results of the course evaluations, as well as those related to competence acquisition among the two first student cohorts (Cohort 1: 2005–2006; Cohort 2: 2006–2007) at the end of the master's training.

## Methods

### Conceptual framework

We used a conceptual framework that bases programme evaluation on four levels of outcomes [23]:

• Level 1: Reaction = participants' satisfaction;

• Level 2: Learning = participants' knowledge acquisition, improved skills or changes in attitude;

• Level 3: Behaviour = changes in participants' on-the-job behaviour;

• Level 4: Results = final change at the organizational and population levels.

Our discussion here is limited to levels 1 to 3.

### Data collection tools

#### Reaction

At the end of each session and course, every student of Cohort 1 completed a standardized questionnaire containing nine closed questions (Likert-type scale of 1 to 5) and one or two open questions.

#### Learning

**Figure 3:**
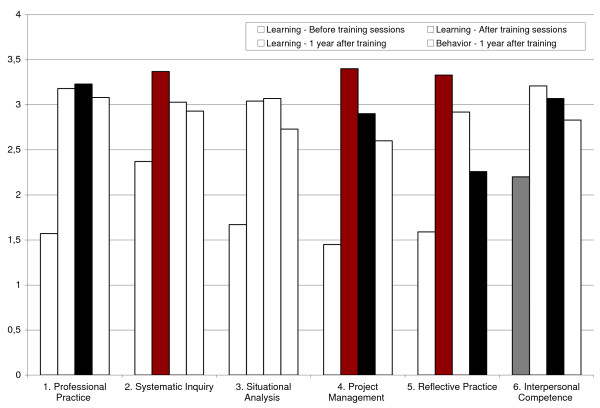
**Mean score for competences cluster for Cohort 1 only**.

We used a standardized questionnaire adapted from the taxonomy of essential competences for programme evaluators [16,17,24]. This taxonomy is a list of 60 competences clustered into six major categories (see Fig. 3), translated into French. As is often the case for this type of evaluation [25], it was impossible to do a pretest before the course because most of the vocabulary was unfamiliar to students. Thus, as has been recommended [25,26], we used a retrospective pretest and post-test. The test was administered at the end of the evaluation course, which also corresponds to the end of the master's programme. In addition, for the first cohort of students (n = 17), a second post-test was administered one year later. For each competence, students were asked to assess, on a Likert-type scale of 1 to 4 (easily ... not at all), their degree of mastery before ("I was able to...") and after ("I am able to...") the master's programme.

#### Behavior

By means of the same questionnaire as for competences, we asked students of Cohort 1 whether they had used them (Likert-type scale of 1 to 4 (easily ... not at all)).

### Data analysis

Programme effects were tested by comparing differences in mean scores between times (before, after, one year after) by pretest-post-test design. Paired sample tests were used to compare mean scores. Data analyses were carried out with SPSS^©^.

## Results

### Participants

Cohort 1 consisted of 17 students: nine men and eight women, from eight West African countries. Cohort 2 was made up of 19 students: 11 men and eight women, from 11 countries. These students come from a wide variety of disciplines: medicine (13), sociology (10), psychology (2), geography/development (5), pharmacy (3), statistics (1), demographics (1) and nutrition (1).

### Trainees' reaction

The evaluation by Cohort 1 of the content of each of the 16 modules of the master's programme is presented in the Additional file 3. Additional file 4 presents the results of the evaluations of each lesson (Table 2) of the evaluation course.

### Trainees' learning

Both cohorts felt they had greatly improved their mastery of the 60 competences by the end of the master's programme. The differences were all positive and all statistically significant for each of the competences (Additional file 5) and for five of the six clusters (Table 4). For both cohorts, the smallest gain was in interpersonal competences, but the level for this before the course was already among the highest (Fig. 3). On the other hand, reflective practice grew substantially in both cohorts.

**Table 4:** Differences in mean scores between points in time for competence clusters

**Cluster of competences**	**Learning**		**Behaviour versus Learning**	
	
	After versus Before		1 year After versus After	1 year After
	
	Cohort 1 (N = 17)	Cohort 2 (N = 19)	Cohort 1 (N = 17)	Cohort 1 (N = 8)
	
	Mean	Mean	Mean	Mean
1. Professional practice	1.61***	1.67***	0.05	-0.25

2. Systematic inquiry	1.00	1.20***	-0.35	-0.11

3. Situational analysis	1.36***	1.54***	0.02	-0.34

4. Project management	1.95**	1.75***	-0.51	-0.33

5. Reflective practice	1.74***	1.99***	-0.42*	-0.41*

6. Interpersonal competence	1.01***	0.97***	-0.15	-0.17

Note: * p < 0.05; ** p < 0.01; *** p < 0.001

Among the 10 competences showing the greatest progression, the four that were common to both cohorts were related to the systematic inquiry cluster (2.6: "specifies programme theory"; 2.8: "develops evaluation designs") and reflective practice (5.3: "pursues professional development in evaluation"; 5.5: "builds professional relationships to enhance evaluation practice"). Among the 10 competences remaining at the lowest level, the five common to both cohorts were related to the systematic inquiry cluster (2.11: "assesses validity of data"; 2.20: "conducts meta-evaluations"); situation analysis (3.5: "addresses conflicts") and project management (4.1: "responds to requests for proposals"; 4.3: "writes formal agreements").

One year after the end of the master's programme, students of Cohort 1 felt that their level of knowledge had been maintained overall, with the exception of reflective practice (Table 4). Detailed analysis of the 60 competences shows a decrease in mastery of five competences after a year (see additional file 3): 4.2 "presents work in a timely manner"; 5.3 "pursues professional development in evaluation"; 5.4 "pursues professional development in relevant content areas"; 5.5 "builds professional relationships to enhance evaluation practice"; 6.6 "demonstrates cross-cultural competence". Three of these competences are located in cluster 5 (reflective practice).

### Trainees' behaviours

Among the 15 students of Cohort 1 who responded to the questionnaire a year later, eight (53%) had carried out evaluations, four (26%) had participated in evaluations, and three (20%) had commissioned evaluations. Students who reported having put their knowledge into practice over the intervening 12 months observed a negative gap between their declared mastery and their actual practice (Table 4, Fig. 3). However, this is statistically significant only for reflective practice. A close look at all 60 competences reveals that the situation is the same for 40 of them, where there is a negative gap between declared mastery and actual practice. However, this gap is statistically significant for only two competences: 3.2: "determines programme evaluability", and 5.1: "aware of self as an evaluator" (see additional file 3).

## Discussion

A number of methodological limitations to the reported results should be mentioned. First, while our assessment was exhaustive, our sample sizes were small, and thus it is quite possible that the difference between behaviour and learning for Cohort 1 is not statistically significant (n = 8 or 7). Second, with respect to the tools, it is possible that a fatigue bias was introduced into the results of the evaluation of all the lessons and courses of Cohort 1. In the African context, where students are rarely asked to evaluate courses and teachers [27], a social desirability bias could also have been introduced. However, if this was the case, it would be true for all the courses and not only for the one described in this article. In addition, we believe we chose the proper instrument because "more than three decades of research on post + retrospective pretest method has unequivocally supported this approach" [25].

Our analysis of the teaching of programme evaluation using the process described above shows that not only was it much appreciated by the students but it also produced positive outcomes. The students gained much knowledge and the degree of mastery of competences was increased and maintained over time. The greatest progress was in competences that were very specific to programme evaluation, as opposed to those in which the students already had attained high levels (systematic inquiry and interpersonal competence). It should nevertheless be noted that the positive effects cannot be attributed solely to the evaluation course, since many other courses in the programme also reinforced certain competences that were on the list of 60. The effect, then, is that of the programme as a whole, which is not a master's degree in evaluation, but rather in population and health. The competences in which the students rated low at the end of the programme were in fact elements that were not addressed in the evaluation course or in the master's programme. That being said, students' low rating of the evaluation of data validity (2.11) should certainly be addressed rapidly by those responsible for the programme.

This double positive effect is definitely attributable in part to the skills-based teaching approach. The training in programme evaluation remained practical, dynamic and respectful of the students. This was not surprising, since most teachers in evaluation espouse this type of interactive teaching [3,12,18,21], which was also observed during an experience in Mali [28]. Rapid integration of the concepts into concrete exercises was an effective strategy, as was the availability of the teaching staff during the lessons. The fact that the difference in knowledge acquisition after the course in the "systematic inquiry" cluster (Table 4) was not statistically significant for Cohort 1 can be explained by: (1) a very elevated pre-course self-evaluation (2.37); (2) a selection of students who had already acquired competences in their training prior to the master's programme; and (3) competences that were interdisciplinary.

With respect to level 3 (learning), the data show that it is more difficult to implement evaluation skills than to understand them. In addition, reflective practice remains the only cluster in which the reduction is statistically significant for levels 2 and 3 one year later, while improvements at the end of the master's programme were the highest (Table 4). Thus, the students learned from this perspective, but it is clear that for them, as for all health professionals [29], reflecting in action is not the easiest thing to do. Many skills cannot be sustainably acquired in a university programme; if evaluators' skills are to improve, they must be put into practice. Also, our results suggest the importance of organizing the field of practice in evaluation with the help, for example, of the AfrEA, which could propose continuing education programmes and support reflective practice.

With regard to modalities for evaluating the students' learning, this study shows the importance of integrating summative evaluation into the learning process. From the beginning of the course, students knew the course content, how they would be evaluated at the end, and on what criteria. Transparency was essential. However, the most helpful aspect was that the knowledge and skills considered indispensable for developing an evaluation plan (as an instrument for evaluating learning) were evaluated (through practical exercises) throughout the course.

The tool for assessing evaluation competences has rarely been used, except by its creator [24]. In this case, we found it very useful for understanding the strengths and weaknesses of the teaching provided. It allowed us to measure the level of students' knowledge as well as those elements where there was still work to be done. However, this tool was developed in North America, and the question of whether African evaluators might not need other specific competences remains to be examined.

## Conclusion

This study shows that skills-based teaching is feasible, much appreciated and well-adapted for a university-based evaluation training programme in a West African context. We highlight the importance of integrating summative evaluation into the learning process. Creating a master's-degree programme in population and health in Africa and providing training in evaluation to high-level health professionals from many countries augurs well for scaling up the practice of evaluation in African health systems. However, this cannot occur without significant investment being made across Africa to develop university-based and professional courses in programme evaluation.

## Competing interests

The authors declare that they have no competing interests.

## Authors' contributions

VR led the study, data collection and analysis, and wrote the first draft. All authors contributed to the study's conception and design and reviewed the final draft. CT did the statistical analysis. VR is a research fellow with the *Fonds pour la Recherche en Santé du Québec (FRSQ)*.

## Supplementary Material

Additional file 1 **Drawing the perception of an evaluation (photo).** Each student must produce a drawing representing his or her perception of the evaluation. Click here for file

Additional file 2 **Graphic representation of the logic of an intervention (photo)**. Each team of students must prepare a graphic representation of the constituent elements of a programme's logic. Click here for file

Additional file 3 **Evaluation of the content of each module by the students of Cohort 1 (n = 17).** Results of the evaluation by Cohort 1 of the content of each of the 16 modules of the master's programme. Click here for file

Additional file 4 **Evaluation of the content of each lesson of the "Evaluation 3.3" module by the students of Cohort 1 (n = 17). **Results of the evaluation by Cohort 1 of the content of each of each lesson of the evaluation lesson. Click here for file

Additional file 5 **Mean differences among the 60 competences for the two cohorts**. Mastery of the 60 competences by the end of the master's programme and a year later. Click here for file
