# Supplementary figures and images for: Programme evaluation training for health professionals in francophone Africa: process, competence acquisition and use
Source: Hum Resour Health. 2009 Jan 15;7:3. doi: 10.1186/1478-4491-7-3 (PMC2647897; doi:10.1186/1478-4491-7-3)

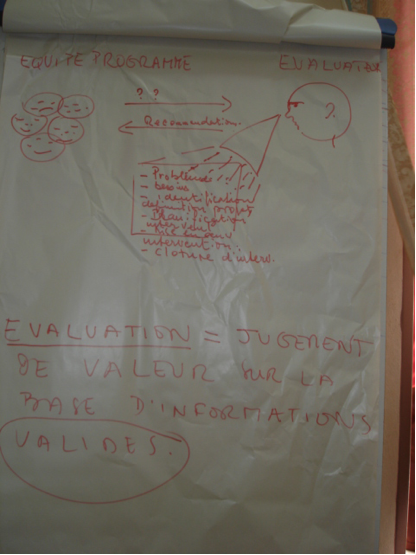

Supplement: Additional file 1 — Drawing the perception of an evaluation (photo). Each student must produce a drawing representing his or her perception of the evaluation. [file 1478-4491-7-3-S1.png]

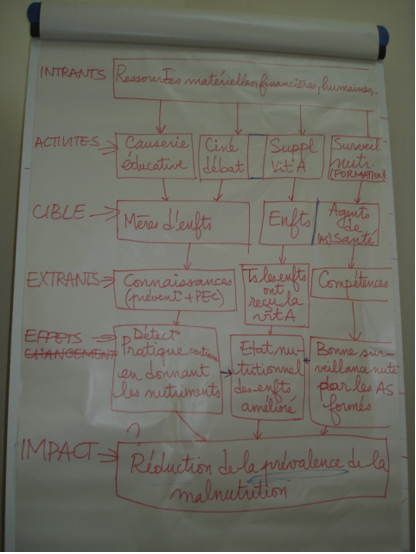

Supplement: Additional file 2 — Graphic representation of the logic of an intervention (photo). Each team of students must prepare a graphic representation of the constituent elements of a programme's logic. [file 1478-4491-7-3-S2.png]
