# Supplementary material for: Programme evaluation training for health professionals in francophone Africa: process, competence acquisition and use
Source: Hum Resour Health. 2009 Jan 15;7:3. doi: 10.1186/1478-4491-7-3 (PMC2647897; doi:10.1186/1478-4491-7-3)
Supplement: Additional file 3 — Evaluation of the content of each module by the students of Cohort 1 (n = 17). Results of the evaluation by Cohort 1 of the content of each of the 16 modules of the master's programme. [file 1478-4491-7-3-S3.doc]

### Additional file 3. Evaluation of the content of each module by the students of Cohort 1 (n=17)

|  | **11** | **12** | **21** | **22** | **23** | **24** | **25** | **31** | **32** | **33** | **41** | **42** | **51** | **52** | **53** | **54** |
| --- | --- | --- | --- | --- | --- | --- | --- | --- | --- | --- | --- | --- | --- | --- | --- | --- |
| Presentation of the module’s plan | 4.65 | 4.53 | 4.00 | 4.00 | 3.43 | 4.12 | 3.82 | 4.06 | 4.27 | 4.46 | 3.56 | 3.69 | 4.25 | 4.47 | 4.06 | 3.94 |
| Planning of the teaching sequence | 4.00 | 4.24 | 3.88 | 3.67 | 2.93 | 4.00 | 3.59 | 4.13 | 4.13 | 4.38 | 3.56 | 3.31 | 4.06 | 4.29 | 4.00 | 3.38 |
| Communicating the learning results to the students | 3.76 | 3.88 | 3.94 | 4.27 | 3.43 | 4.18 | 3.59 | 4.00 | 4.00 | 4.31 | 3.75 | 3.38 | 4.06 | 4.29 | 3.94 | 3.69 |
| Steering the learning situations | 3.94 | 3.88 | 4.00 | 3.87 | 3.43 | 4.12 | 3.41 | 3.94 | 4.00 | 4.38 | 3.81 | 3.31 | 4.25 | 4.29 | 3.94 | 3.38 |
| Appropriateness of evaluation/ content and learning format | 3.94 | 3.35 | 4.12 | 3.60 | 3.29 | 4.12 | 3.59 | 4.00 | 4.13 | 4.38 | 3.93 | 3.50 | 3.94 | 4.18 | 3.53 | 3.69 |
| Presentation of the criteria for correcting exams and student work | 3.65 | 3.59 | 3.80 | 3.50 | 3.21 | 3.69 | 3.31 | 3.53 | 3.62 | 4.46 | 3.56 | 3.67 | 4.00 | 4.00 | 3.88 | 3.75 |
| Coverage of the important aspects of the module in the exams | 4.13 | 3.82 | 3.93 | 4.14 | 3.57 | 4.06 | 3.76 | 3.88 | 4.00 | 4.46 | 3.75 | 3.63 | 4.25 | 4.18 | 3.75 | 3.88 |
| Coverage of the essential aspects of the module | 4.41 | 4.20 | 4.06 | 4.07 | 3.77 | 4.12 | 3.75 | 4.31 | 4.08 | 4.42 | 3.94 | 2.94 | 4.25 | 4.29 | 4.12 | 4.00 |
| Absence of superfluous elements | 3.88 | 3.47 | 3.87 | 4.36 | 3.58 | 4.00 | 3.69 | 4.08 | 4.36 | 4.17 | 3.73 | 3.13 | 4.27 | 4.36 | 3.86 | 3.71 |
| **Overall average** | **4.04** | **3.88** | **3.96** | **3.94** | **3.40** | **4.04** | **3.61** | **3.99** | **4.07** | **4.38** | **3.73** | **3.39** | **4.15** | **4.26** | **3.90** | **3.71** |

Note: See Table 1 for course titles.
