# Supplementary material for: Programme evaluation training for health professionals in francophone Africa: process, competence acquisition and use
Source: Hum Resour Health. 2009 Jan 15;7:3. doi: 10.1186/1478-4491-7-3 (PMC2647897; doi:10.1186/1478-4491-7-3)
Supplement: Additional file 4 — Evaluation of the content of each lesson of the "Evaluation 3.3" module by the students of Cohort 1 (n = 17). Results of the evaluation by Cohort 1 of the content of each of each lesson of the evaluation lesson. [file 1478-4491-7-3-S4.doc]

### Additional file 4. Evaluation of the content of each lesson of the “Evaluation 3.3” module by the students of Cohort 1 (n=17)

|  | **331** | **332** | **333** | **334** | **335** | **336** | **337** | **338** | **339** | **3310** | **3311** | **3312** | **3313** | **Mean** |
| --- | --- | --- | --- | --- | --- | --- | --- | --- | --- | --- | --- | --- | --- | --- |
| Presentation of the lesson plan | 4.29 | 4.35 | 4.12 | 4.12 | 4.18 | 4.25 | 4.24 | 4.35 | 4.35 | 4.33 | 4.25 | 4.18 | 4.25 | **4.25** |
| Use of learning activities | 4.29 | 4.29 | 4.19 | 4.12 | 4.24 | 4.38 | 4.29 | 4.35 | 4.41 | 4.33 | 4.25 | 4.18 | 4.25 | **4.27** |
| Use of learning situations | 4.18 | 4.24 | 4.20 | 4.12 | 4.24 | 4.38 | 4.24 | 4.35 | 4.35 | 4.33 | 4.25 | 4.18 | 4.25 | **4.25** |
| Choice of readings | 4.24 | 4.35 | 4.19 | 4.18 | 4.24 | 4.38 | 4.12 | 4.35 | 4.35 | 4.33 | 4.25 | 4.18 | 4.31 | **4.27** |
| Planning of individual work loads | 4.24 | 4.18 | 4.19 | 4.18 | 4.29 | 4.31 | 4.18 | 4.29 | 4.35 | 4.33 | 4.31 | 4.18 | 4.25 | **4.25** |
| Mastery of the material being taught | 4.59 | 4.47 | 4.44 | 4.41 | 4.47 | 4.56 | 4.53 | 4.59 | 4.59 | 4.60 | 4.44 | 4.29 | 4.50 | **4.50** |
| Responses to students’ questions | 4.41 | 4.35 | 4.19 | 4.35 | 4.35 | 4.44 | 4.35 | 4.41 | 4.47 | 4.47 | 4.25 | 4.18 | 4.37 | **4.35** |
| Respect for students’ ideas | 4.35 | 4.41 | 4.25 | 4.24 | 4.35 | 4.44 | 4.35 | 4.47 | 4.47 | 4.40 | 4.31 | 4.24 | 4.37 | **4.36** |
| Availability of learning resources | 4.18 | 4.24 | 4.06 | 4.18 | 4.18 | 4.38 | 4.29 | 4.41 | 4.41 | 4.40 | 4.31 | 4.24 | 4.31 | **4.28** |
| **Average** | **4.31** | **4.32** | **4.20** | **4.21** | **4.28** | **4.39** | **4.29** | **4.40** | **4.42** | **4.39** | **4.29** | **4.21** | **4.32** | **4.31** |

Note: See Table 2 for lesson titles.
