# Supplementary material for: Programme evaluation training for health professionals in francophone Africa: process, competence acquisition and use
Source: Hum Resour Health. 2009 Jan 15;7:3. doi: 10.1186/1478-4491-7-3 (PMC2647897; doi:10.1186/1478-4491-7-3)
Supplement: Additional file 5 — Mean differences among the 60 competences for the two cohorts. Mastery of the 60 competences by the end of the master's programme and a year later. [file 1478-4491-7-3-S5.pdf]

ANNEX TABLE: RESULTS ON EVALUATION OF COMPETENCIES - COHORT 1 AND COHORT 2

		LEARNING												BEHAVIOR vs LEARNING			
items no.	LISTE des COMPETENCES	Before - After the training sessions								1 year after - After training sessions				1 year after			
		COHORT 1				COHORT 2				COHORT 1				COHORT 1			
		N	Mean	Std dev	p	N	Mean	Std dev	p	N	Mean	Std dev	p	N	Mean	Std dev	p
**1.0**	**Professional Practice**																
1.1	Applies professional evaluation standards	16	1,50	0,516	0,000	19	1,79	0,419	0,000	13	0,31	0,630	0,104	8	-0,38	0,744	0,197
1.2	Acts ethically and strives for integrity and honesty in conducting evaluations	16	1,50	0,816	0,000	19	1,53	0,612	0,000	13	-0,23	0,725	0,273	8	0,13	0,354	0,351
1.3	Conveys personal evaluation approaches and skills to potential clients	17	1,82	0,636	0,000	19	1,68	0,671	0,000	14	0,00	0,679	1,000	8	-0,13	0,354	0,351
1.4	Respects clients, respondents, program participants, and other stakeholders	16	1,56	0,814	0,000	19	1,26	1,195	0,000	14	0,00	0,679	1,000	t cannot be computed because the standard error of the difference is 0.			
1.5	Considers the general and public welfare in evaluation practice	15	1,47	0,743	0,000	19	1,74	0,933	0,000	12	0,00	0,426	1,000	8	-0,63	1,061	0,140
1.6	Contributes to the knowledge base of evaluation	15	1,80	0,561	0,000	19	2,00	0,577	0,000	12	0,08	0,515	0,586	8	-0,50	0,926	0,170

ANNEX TABLE: RESULTS ON EVALUATION OF COMPETENCIES - COHORT 1 AND COHORT 2

		LEARNING												BEHAVIOR vs LEARNING			
items no.	LISTE des COMPETENCES	Before - After the training sessions								1 year after - After training sessions				1 year after			
		COHORT 1				COHORT 2				COHORT 1				COHORT 1			
		N	Mean	Std dev	p	N	Mean	Std dev	p	N	Mean	Std dev	p	N	Mean	Std dev	p
**2.0**	**Systematic Inquiry**																
2.1	Understands the knowledge base of evaluation (terms, concepts, theories, assumptions)	17	1,82	0,529	0,000	19	1,74	0,806	0,000	15	0,07	0,704	0,719	8	-0,13	0,354	0,351
2.2	Knowledgeable about quantitative methods	17	1,29	0,588	0,000	19	1,05	0,524	0,000	14	0,21	0,802	0,336	7	-0,29	0,488	0,172
2.3	Knowledgeable about qualitative methods	17	1,12	0,600	0,000	19	1,26	0,733	0,000	14	-0,07	0,829	0,752	8	0,25	0,463	0,170
2.4	Knowledgeable about mixed methods	16	1,31	0,479	0,000	19	1,42	0,692	0,000	13	0,08	0,760	0,721	8	0,13	0,641	0,598
2.5	Conducts literature reviews	17	1,41	0,618	0,000	19	1,00	0,745	0,000	14	-0,36	0,742	0,136	7	-0,29	0,488	0,172
2.6	Specifies program theory	16	2,06	0,443	0,000	19	1,84	0,501	0,000	14	-0,21	0,802	0,336	t cannot be computed because the standard error of the difference is 0.			
2.7	Frames evaluation questions	17	1,88	0,600	0,000	19	1,79	0,419	0,000	15	0,07	0,594	0,670	8	-0,13	0,354	0,351

ANNEX TABLE: RESULTS ON EVALUATION OF COMPETENCIES - COHORT 1 AND COHORT 2

items no.	LISTE des COMPETENCES	LEARNING												BEHAVIOR vs LEARNING			
		Before - After the training sessions								1 year after - After training sessions				1 year after			
		COHORT 1				COHORT 2				COHORT 1				COHORT 1			
		N	Mean	Std dev	p	N	Mean	Std dev	p	N	Mean	Std dev	p	N	Mean	Std dev	p
2.8	Develops evaluation designs	17	1,94	0,659	0,000	19	1,95	0,621	0,000	15	-0,20	0,676	0,271	8	-0,13	0,354	0,351
2.9	Identifies data sources	16	1,25	0,931	0,000	19	1,26	0,806	0,000	15	-0,07	0,458	0,582	7	-0,14	0,378	0,356
2.10	Collects data	17	1,06	0,827	0,000	19	0,89	0,658	0,000	15	-0,07	0,704	0,719	8	-0,13	0,354	0,351
2.11	Assesses validity of data	17	1,47	0,514	0,000	19	1,16	0,765	0,000	15	-0,07	0,704	0,719	8	0,00	0,535	1,000
2.12	Assesses reliability of data	17	1,29	0,849	0,000	19	1,11	0,658	0,000	15	-0,20	0,676	0,271	8	-0,25	0,463	0,170
2.13	Analyzes data	16	1,13	0,719	0,000	19	1,00	0,667	0,000	15	-0,13	0,352	0,164	8	0,00	0,535	1,000
2.14	Interprets data	14	0,93	0,616	0,000	19	0,89	0,658	0,000	14	-0,07	0,267	0,336	8	-0,13	0,354	0,351
2.15	Makes judgements	17	1,06	0,827	0,000	19	1,11	0,658	0,000	15	0,13	0,516	0,334	8	-0,13	0,354	0,351

ANNEX TABLE: RESULTS ON EVALUATION OF COMPETENCIES - COHORT 1 AND COHORT 2

items no.	LISTE des COMPETENCES	LEARNING												BEHAVIOR vs LEARNING			
		Before - After the training sessions								1 year after - After training sessions				1 year after			
		COHORT 1				COHORT 2				COHORT 1				COHORT 1			
		N	Mean	Std dev	p	N	Mean	Std dev	p	N	Mean	Std dev	p	N	Mean	Std dev	p
2.16	Develops recommendations	17	1,35	0,702	0,000	19	1,11	0,459	0,000	15	-0,13	0,640	0,433	8	-0,13	0,354	0,351
2.17	Provides rationales for decisions throughout the evaluation	17	1,71	0,772	0,000	19	1,68	0,582	0,000	15	-0,40	0,910	0,111	7	-0,14	0,690	0,604
2.18	Reports evaluation procedures and results	15	1,73	0,704	0,000	19	1,74	0,562	0,000	12	-0,17	0,389	0,166	t cannot be computed because the standard error of the difference is 0.			
2.19	Notes strengths and limitations of the evaluation	16	1,63	0,500	0,000	19	1,74	0,562	0,000	15	-0,27	0,594	0,104	8	-0,13	0,354	0,351
2.20	Conducts meta-evaluations	15	0,93	0,704	0,000	19	1,42	0,607	0,000	13	-0,08	0,760	0,721	8	-0,50	0,929	0,170

ANNEX TABLE: RESULTS ON EVALUATION OF COMPETENCIES - COHORT 1 AND COHORT 2

		LEARNING												BEHAVIOR vs LEARNING			
items no.	LISTE des COMPETENCES	Before - After the training sessions								1 year after - After training sessions				1 year after			
		COHORT 1				COHORT 2				COHORT 1				COHORT 1			
		N	Mean	Std dev	p	N	Mean	Std dev	p	N	Mean	Std dev	p	N	Mean	Std dev	p
3.0	Situational Analysis																
3.1	Describes the program	16	1,63	0,719	0,000	19	1,58	1,071	0,000	15	0,07	0,594	0,670	8	0,00	0,535	1,000
3.2	Determines program evaluability	17	1,59	0,618	0,000	19	1,84	0,688	0,000	15	0,00	0,655	1,000	8	-0,75	0,886	0,048
3.3	Identifies the interests of relevant stakeholders	16	1,44	0,814	0,000	19	1,74	0,806	0,000	15	0,13	0,516	0,334	8	-0,63	1,061	0,140
3.4	Serves the information needs of intended users	17	1,53	0,717	0,000	19	1,53	0,841	0,000	15	-0,07	0,704	0,719	8	-0,13	0,354	0,351
3.5	Addresses conflicts	17	1,18	0,529	0,000	19	1,05	0,780	0,000	15	-0,07	0,704	0,719	8	-1,00	1,309	0,068
3.6	Examines the organizational context of the evaluation	16	1,38	0,500	0,000	19	1,74	0,653	0,000	14	-0,07	0,616	0,671	8	-0,50	1,195	0,275
3.7	Analyzes the political considerations relevant to the evaluation	17	1,41	0,618	0,000	19	1,68	0,582	0,000	15	0,00	0,655	1,000	8	-0,38	0,916	0,285

ANNEX TABLE: RESULTS ON EVALUATION OF COMPETENCIES - COHORT 1 AND COHORT 2

		LEARNING												BEHAVIOR vs LEARNING			
items no.	LISTE des COMPETENCES	Before - After the training sessions								1 year after - After training sessions				1 year after			
		COHORT 1				COHORT 2				COHORT 1				COHORT 1			
		N	Mean	Std dev	p	N	Mean	Std dev	p	N	Mean	Std dev	p	N	Mean	Std dev	p
3.8	Attends to issues of evaluation use	16	1,44	0,512	0,000	19	1,53	0,772	0,000	15	-0,07	0,594	0,670	8	-0,13	0,354	0,351
3.9	Attends to issues of organizational change	16	1,31	0,602	0,000	19	1,63	0,684	0,000	14	0,14	0,535	0,336	8	-0,50	0,756	0,104
3.10	Respects the uniqueness of the evaluation site and client	17	1,59	0,618	0,000	19	1,95	0,780	0,000	15	0,20	0,676	0,271	8	0,00	0,535	1,000
3.11	Remains open to input from others	17	0,94	0,659	0,000	19	1,00	0,943	0,000	15	-0,13	0,640	0,433	8	0,00	0,535	1,000
3.12	Modifies the study as needed	17	0,94	0,659	0,000	19	1,26	0,806	0,000	15	-0,07	0,799	0,751	8	-0,13	0,354	0,351

ANNEX TABLE: RESULTS ON EVALUATION OF COMPETENCIES - COHORT 1 AND COHORT 2

		LEARNING												BEHAVIOR vs LEARNING			
items no.	LISTE des COMPETENCES	Before - After the training sessions								1 year after - After training sessions				1 year after			
		COHORT 1				COHORT 2				COHORT 1				COHORT 1			
		N	Mean	Std dev	p	N	Mean	Std dev	p	N	Mean	Std dev	p	N	Mean	Std dev	p
**4.0**	**Project Management**																
4.1	Responds to requests for proposals	16	1,44	0,512	0,000	19	1,63	0,597	0,000	14	0,00	0,555	1,000	8	-0,38	0,744	0,197
4.2	Negotiates with clients before the evaluation begins	17	1,53	0,624	0,000	19	1,68	0,582	0,000	15	-0,20	0,775	0,334	8	-0,38	0,744	0,197
4.3	Writes formal agreements	16	1,38	0,619	0,000	19	1,58	0,769	0,000	14	-0,07	0,730	0,720	8	-0,50	0,926	0,170
4.4	Communicates with clients throughout the evaluation process	16	1,50	0,966	0,000	19	1,95	0,524	0,000	14	-0,36	0,745	0,096	8	-0,25	0,707	0,351
4.5	Budgets an evaluation	17	1,47	0,800	0,000	19	1,79	0,535	0,000	15	-0,13	0,743	0,499	8	-0,38	0,744	0,197
4.6	Justifies cost given information needs	17	1,47	0,717	0,000	19	1,84	0,501	0,000	15	-0,13	0,743	0,499	8	-0,50	0,756	0,104
4.7	Identifies needed resources for evaluation, such as information, expertise, personnel, instruments	17	1,65	0,702	0,000	19	1,79	0,535	0,000	15	-0,07	0,594	0,670	8	0,00	0,535	1,000

ANNEX TABLE: RESULTS ON EVALUATION OF COMPETENCIES - COHORT 1 AND COHORT 2

		LEARNING												BEHAVIOR vs LEARNING			
items no.	LISTE des COMPETENCES	Before - After the training sessions								1 year after - After training sessions				1 year after			
		COHORT 1				COHORT 2				COHORT 1				COHORT 1			
		N	Mean	Std dev	p	N	Mean	Std dev	p	N	Mean	Std dev	p	N	Mean	Std dev	p
4.8	Uses appropriate technology	15	1,40	0,632	0,000	19	1,74	0,452	0,000	14	0,00	0,679	1,000	t cannot be computed because the standard error of the difference is 0.			
4.9	Supervises others involved in conducting the evaluation	16	1,50	0,816	0,000	19	1,68	0,478	0,000	15	0,13	0,640	0,433	8	-0,50	0,926	0,170
4.10	Trains others involved in conducting the evaluation	16	1,31	0,479	0,000	19	1,84	0,602	0,000	14	0,00	0,784	1,000	7	-0,71	0,951	0,094
4.11	Conducts the evaluation in a nondisruptive manner	17	1,35	0,702	0,000	19	2,00	0,471	0,000	14	0,00	0,679	1,000	t cannot be computed because the standard error of the difference is 0.			
4.12	Presents work in a timely manner	14	1,50	0,519	0,000	19	1,53	0,697	0,000	13	-0,38	0,506	0,018	8	-0,29	0,488	0,172

ANNEX TABLE: RESULTS ON EVALUATION OF COMPETENCIES - COHORT 1 AND COHORT 2

		LEARNING												BEHAVIOR vs LEARNING			
items no.	LISTE des COMPETENCES	Before - After the training sessions								1 year after - After training sessions				1 year after			
		COHORT 1				COHORT 2				COHORT 1				COHORT 1			
		N	Mean	Std dev	p	N	Mean	Std dev	p	N	Mean	Std dev	p	N	Mean	Std dev	p
**5.0**	**Reflective Practice**																
5.1	Aware of self as an evaluator (knowledge, skills, dispositions)	16	1,50	0,816	0,000	19	1,95	0,780	0,000	14	-0,29	0,825	0,218	7	-0,71	0,756	0,047
5.2	Reflects on personal evaluation practice (competencies and areas for growth)	16	1,63	0,719	0,000	19	2,00	0,882	0,000	14	-0,36	1,008	0,208	7	-0,43	0,787	0,200
5.3	Pursues professional development in evaluation	17	2,00	0,866	0,000	19	2,37	0,597	0,000	14	-0,50	0,760	0,029	7	-0,43	0,787	0,200
5.4	Pursues professional development in relevant content areas	16	1,50	0,730	0,000	19	1,63	0,684	0,000	13	-0,46	0,660	0,027	6	-0,17	0,408	0,363
5.5	Builds professional relationships to enhance evaluation practice	16	1,94	0,680	0,000	19	2,00	0,745	0,000	14	-0,64	1,008	0,033	7	0,14	0,900	0,689

ANNEX TABLE: RESULTS ON EVALUATION OF COMPETENCIES - COHORT 1 AND COHORT 2

		LEARNING												BEHAVIOR vs LEARNING			
items no.	LISTE des COMPETENCES	Before - After the training sessions								1 year after - After training sessions				1 year after			
		COHORT 1				COHORT 2				COHORT 1				COHORT 1			
		N	Mean	Std dev	p	N	Mean	Std dev	p	N	Mean	Std dev	p	N	Mean	Std dev	p
**6.0**	**Interpersonal Competence**																
6.1	Uses written communication skills	15	73,00	0,799	0,000	19	0,84	0,834	0,000	14	0,14	0,663	0,435	t cannot be computed because the standard error of the difference is 0.			
6.2	Uses verbal/listening communication skills	14	0,79	0,802	0,003	19	0,84	0,688	0,000	14	0,07	0,829	0,752	t cannot be computed because the standard error of the difference is 0.			
6.3	Uses negotiation skills	16	1,00	0,730	0,003	19	1,00	0,667	0,000	15	0,20	0,561	0,189	t cannot be computed because the standard error of the difference is 0.			
6.4	Uses conflict resolution skills	14	0,93	0,616	0,000	19	0,89	0,658	0,000	14	-0,29	0,726	0,165	8	-0,38	0,916	0,285
6.5	Facilitates constructive interpersonal interaction (teamwork, group facilitation, processing)	14	1,00	0,555	0,000	19	1,11	0,658	0,000	15	-0,27	0,594	1,040	8	-0,25	0,707	0,351
6.6	Demonstrates cross-cultural competence	16	1,13	0,619	0,000	19	1,16	0,834	0,000	15	-0,33	0,488	0,019	8	-0,38	0,744	0,197
